# Adaptive preprocessing and Cascaded Canny Edge Segmentation for cassava disease identification using HyperCapsInception-ResNet-V2-CNN

**DOI:** 10.3389/fpls.2025.1701030

**Published:** 2025-12-19

**Authors:** Sathishkumar M, Geetha K

**Affiliations:** Department of Computer Science and Engineering, Excel Engineering College, Namakkal, Tamil Nadu, India

**Keywords:** cassava disease, feature optimization, classification, enhanced contrast, segmentation, affected region, deep learning

## Abstract

**Introduction:**

Cassava is one of the most widely cultivated crops worldwide, renowned for its rich natural ingredients and numerous nutritional benefits. However, the complex interdependencies among its features often pose challenges in image restoration and segmentation, particularly when identifying disease regions. In previous work, this manifested as higher false positives and misidentification of non-relevant areas, leading to a decline in precision and accuracy.

**Methods:**

To address these issues, this study proposed an efficient artificial intelligence-powered image analysis system that leverages optimal feature selection with a HyperCapsInception-ResNet-V2-CNN model to enhance disease detection accuracy. Initially, the dataset was collected from the Kaggle repository, its name was Cassava Leaf Disease Classification, and it comprised 21,367 different images. Our approach began by normalizing cassava plant disease data using adaptive Gaussian Otsu thresholding. Histogram color evaluation and iterative clustering fragmentation were then applied to better isolate disease variations and improve precision. Subsequently, Cascaded Canny Edge Segmentation (CCES) was used to effectively segment the disease region. The disease variation properties were further evaluated using the Optimal Spider Swarm Intelligence Technique (OSSIT) to reduce irrelevant feature dimensions. For classification, the HyperCapsInception-ResNet-V2-CNN model was employed to categorize cassava diseases, including cassava bacterial blight (CBB), cassava mosaic disease (CMD), cassava green mite (CGM) disease, and cassava brown streak disease (CBSD), along with regular and abnormal leaf states.

**Results:**

The proposed method’s simulation results achieved 98.15% accuracy, a 97.22% F1-score, and 96.02% precision, outperforming other traditional methods such as EfficientNetB3, AlexNet, Faster-RCNN, and InceptionV3.

**Discussion:**

Both optimized feature selection with OSSIT and hybrid HyperCapsInception-ResNet-V2-CNN architecture significantly enhanced the detection reluctance and the classification of the data. These findings indicate that the proposed system is effective in the automated detection of cassava disease and has a high potential of being practical in agricultural practices especially in precision farming and early detection of diseases.

## 1 Introduction

Agriculture serves as the backbone of economic development and is essential to driving global food production through one of the largest supply chains in commercial agriculture. Regular evaluations of biotechnological solutions for plant leaf diseases, particularly those affecting cassava, are crucial for addressing challenges in disease identification and the application of modern technologies. These assessments play a vital role in enhancing cassava farming by addressing critical constraints such as nutrient deficiencies and disease susceptibility. Modern tools, including genomics, genome-assisted breeding, molecular techniques, and gene-editing technologies, have significantly improved the productivity and resilience of economically important crops such as cassava (Otun et al., 2023). Cassava bacterial blight (CBB), cassava mosaic disease (CMD), cassava green mite (CGM) disease, and cassava brown streak disease (CBSD), which are caused by a complex of soil-borne pathogens, exemplify the devastating impact of diseases on cassava. These can lead to total crop failure in susceptible cultivars (Onofre et al., 2024). The primary propagation method of using cassava stem cuttings from local farms further exacerbates disease spread, especially viral infections, due to contaminated or low-quality planting materials (Ntui et al., 2024).

Cassava’s vulnerability to leaf diseases is profound, with yield losses reaching up to 95% under severe conditions. Traditional local varieties are particularly susceptible. Globally, major diseases such as CMD and CBB are aggravated by factors including the reuse of infected planting materials, the cultivation of disease-prone varieties, and efficient insect vectors. The severity of these diseases depends on infection duration, virulence, and interactions among viral species, crop age, and host sensitivity (Raju et al., 2023).

Recent advances in plant disease detection, powered by deep learning, offer promising solutions. However, these techniques often only perform optimally under specific conditions, and early detection remains a challenge. Integrating time-series datasets can address these limitations. Furthermore, cassava flour production generates a nutrient-rich, yellowish liquid waste that has potential for biotechnological applications. Strategies such as pathogen-derived resistance (PDR), which utilize viral proteins such as movement protein (MP), coat protein (CP), and replicase protein (RP), also offer innovative approaches for combating viral diseases (Xu et al., 2024).

Despite numerous studies examining cassava leaf disease detection methods based on traditional image processing and deep learning, this field still faces several limitations. Current methods often lack feature discrimination, leading to false positives and poor classification when similar disease symptoms and complex leaf textures are present (Karthik et al., 2024). A variety of models leverage handcrafted features or convolutional neural network (CNN) architectures based on shallow learning, which have limited capacity to learn multi-scale spatial and contextual data. Moreover, unequal image quality, lighting variations, and background noise are causing additional problems in robust segmentation and feature extraction. Furthermore, inadequate comparative validation, small or skewed datasets, and the absence of optimized feature selection schemes have limited the generalization and accuracy of models.

The classification of cassava leaf diseases relies primarily on labeled data. The HyperCapsInception-ResNet-V2-CNN model has emerged as an effective tool for this purpose, combining advanced connection patterns and feature extraction techniques to achieve high classification accuracy. The Residual Network (ResNet) component uses residual connections to enhance gradient flow during training, addressing challenges such as vanishing gradients and accelerating convergence. ResNet-based architectures have achieved classification accuracies of 92%–98%, making them particularly effective for agricultural diagnostics (Choudhury et al., 2025).

Cassava, a staple crop in many regions, faces significant challenges from diseases and nutritional deficiencies. The symptoms of cassava leaf diseases, such as blight, withering, necrosis, and dieback, are readily observable. For example, CGM is caused by mites that attack immature leaves, destroying their contents with piercing mouthparts. Another major threat, CMD, is caused by viruses and manifests in severe symptoms in infected plants. Cassava’s inherent nutritional limitations, such as low protein content and insufficient levels of vitamins A and E, iron, and zinc, further exacerbate its vulnerability. These deficiencies pose serious concerns in malnutrition-prone regions where cassava serves as a dietary staple (Akinpelu et al., 2025).

This challenge is addressed by proposing the HyperCapsInception-ResNet-V2-CNN model for accurate cassava disease classification. By leveraging feature selection techniques, the model enables timely and precise disease detection, preserves crop health, enhances food security, and promotes sustainable agricultural practices. Modern technological advancements, such as deep learning, have significantly enhanced plant disease detection and classification (Sneha Snigdha et al., 2025).

Deep ResNets excel at identifying plant leaf diseases because they can process complex visual features. High-resolution images of cassava leaves are fed into a pre-trained or custom-designed ResNet model, where convolutional layers extract hierarchical features such as texture, color, and patterns. Residual blocks enable the network to learn both high-level and low-level representations. The extracted features are then passed through fully connected layers for disease classification. Transfer learning and fine-tuning further improve accuracy, even with limited labeled data, making this approach suitable for real-world applications (Alabi. et al., 2024) (Pandey and Ramesh, 2024),.

The classification of cassava leaves affected by CMD and CBSD, two prevalent cassava disorders, demonstrates significant advancements in disease detection. By employing Generalized Matrix Relevance Learning Vector Quantization, prediction accuracies of 95.1% for healthy samples, 75.9% for CBSD, and 85.7% for CMD were achieved. These outcomes surpass those of traditional classification methods, highlighting the potential for early and accurate disease detection (Owomugisha et al., 2021). This system not only informs farmers about field health and disease spread but also enables them to take preventive measures that enhance crop productivity. Additionally, integrating mobile applications connects farmers with plant pathology experts and disease databases, fostering broader community benefits (Coletta, 2020).

The Swin Transformer uses self-attention and shifting windows to capture global features. At the same time, the Dual-Attention Multi-scale Fusion Network (DAMFN) incorporates Multi-Separable Attention (MSA) and Tri-Shuffle Convolution Attention (TSCA) units for fine-grained feature extraction. These advanced architectures achieve an impressive accuracy of 95.68%, surpassing state-of-the-art methods (Dhiyanesh et al., 2025). Complementing these efforts, the fuzzification process maps environmental parameters related to cassava production into membership functions for low, medium, and high ranges, refining data representation for predictive models (Iklima, 2024).

K-means clustering has proven effective for identifying cassava diseases by locating cluster centers and segmenting diseased areas. This process, combined with the AlexNet model and Support Vector Machine (SVM) and K-nearest neighbor (KNN) classifiers, enhanced by data augmentation, has resulted in robust metrics, including 90.7% accuracy, an 83.5% F1-score, 83.5% sensitivity, and 93.7% specificity (Sholihin et al., 2023). Addressing mixed infections of Cassava Brown Streak Virus (CBSV), numerical models demonstrate that the early management of the whitefly vector significantly reduces crop losses and disease prevalence, safeguarding food security in East and Central Africa (Dhiyanesh et al., 2024).

Thermal imaging techniques have also been explored to monitor cassava tuber deterioration. Between days 0 and 30, tubers were categorized into three degradation levels using classifiers such as KNN, decision trees, and ensemble models. Among these, Linear Discriminant Analysis (LDA), SVM, and ensemble classifiers achieved the highest accuracy of 86.7%, effectively distinguishing degradation stages (Posom et al., 2023). However, achieving a balance between accuracy and computational complexity in large-scale farming remains a challenge. MobileNet, for example, achieves 88.28% accuracy while maintaining low complexity, making it a promising solution for efficient disease detection (Abishek et al., 2023).

The integration of multiple datasets into a unified dataset has further improved feature extraction and model generalization. An enhanced MobileNet model, scalable to 64 classes across 22 crop sets, achieved an accuracy of 95.94% (Tembhurne et al., 2023). Moreover, a web-based application built on an MongoDB, Express, React, and Node. Js (MERN) architecture facilitated disease prediction and treatment using a pre-trained convolutional neural network. This integrated approach demonstrates the effectiveness of unified datasets for robust feature extraction and improved user accessibility (Khan and Srivastava, 2023).

For cassava mosaic disease management, a three-step approach has been outlined. First, it involves comparing 166 model disease risks based on environmental variables. Second, it estimates the disease spread network using data on planting materials. Finally, analytical methods such as partial least squares regression (PLSR) and K-nearest neighbor regression (KNNR) are applied. Among these, CovSel_MLR delivered the best performance, recording an optimism coefficient, a root mean square error of prediction (RMSEP) of 0.96 g/100 g, and a ratio of standard deviation (RPD) of 3.60 for prediction (Meghar et al., 2024).

To detect apple leaf diseases such as scab and black rot, researchers utilized raw input images from the PlantVillage collection to segment affected areas. This technique significantly improved performance indicators, increasing the F1-score from 31% to 38%, accuracy from 19% to 29%, precision from 19% to 28%, and sensitivity from 31% to 38% (Allaoua Chelloug et al., 2023). Similar techniques have been used for corn, coffee, strawberries, and grapes. High-performance models, such as the Cascading Autoencoder and Attention Residual U-Net (CAAR-UNet), produced remarkable outcomes, including a mean pixel accuracy of 95.26% and a weighted mean intersection over union (wm-IoU) of 0.7451. These architectures give crop disease detection a competitive edge (Abinaya et al., 2023).

In another development, the Swin Rout, a variant of the Swin Transformer, utilizes multi-level structured designs for visual feature extraction, achieving 4.53% and 1.81% improvements over popular models. With a top-1 accuracy of 82.19% and an F1-score of 82.79%, it has demonstrated its robustness in agricultural disease classification (Li et al., 2024). Lightweight networks and transfer learning techniques have been effectively used to classify soybean leaf diseases. The fuzzy ensemble technique enhanced precision and accuracy, while the CycleGAN network achieved 94.27% identification accuracy and an average F1-score of 94% (Hang et al., 2024).

Similarly, CAR-Caps Net has excelled at detecting nutrient deficiencies in rice crops, achieving 97.1% accuracy. It outperformed VGG19 (91.8%), SVM with C-means clustering (92%), and Random Forest Regression (81.82%) (Amudha and Brindha, 2024). Comparisons of lightweight models for maize leaf disease diagnosis revealed that while MobileNetV2 achieved 92.48% accuracy with a loss of 0.19020; EfficientNetB3 outperformed it with 93.20% accuracy and a loss of 0.0850. Despite MobileNetV2’s faster computational speed (Naveenkumar et al., 2022), EfficientNetB3’s advanced architecture and compound scaling demonstrated superior efficiency (Riyadi et al., 2024). **Table 1** describes the traditional methods for identifying cassava leaf disease.

**Table 1:** Traditional methods for cassava leaf disease identification.

References	Algorithm used	Output	Dataset	Limitation
(Boonprong et al., 2024)	Bare Land Referenced Algorithm from Hyper-Temporal Data (BRAH)	Accuracy 94%	Landsat satellite images	Limited to specific hyper-temporal datasets, with potential for overfitting
(Emmanuel et al., 2023)	VGG16 Model Hybrid	Accuracy 88.6%, precision 88.4%	Cassava leaves dataset	Highly computational, prone to overfitting with small datasets
(Yuceturk and Eren, 2023)	Field (Non-PCA)—Spectral. XGBoost	Accuracy 85%, precision 85%	Cassava leaves dataset	Requires large datasets for optimal performance
(Singh, 2023)	Inception-ResNet-V2	Accuracy 87%,	Cassava leaves dataset	High processing requirements and complex training
(Singhpoo et al., 2023)	Yolo v4 model Mask R-CNN	Average error 3.50%	Cassava leaves dataset	Implementing for real-time applications requires fine-tuning
(Matesun and Makolo, 2023)	Random Forest (RF)	Accuracy 56%	Hygienic cassava dataset	Limited accuracy, not suitable for high-dimensional data
(Chilakalapudi and Jayachandran, 2024)	AlexNet	Precision, 91% as recall, and 91% as F1-score	PlantVillage Dataset	An error occurs during testing
(Chai et al., 2024)	EfficientNetB1, DenseNet121	Accuracy by 2.13% to 3.06%	Cassava 2020 dataset	Suffered major crop losses
(Keerthana et al., 2024)	SEResNext50 32x4d model	Accuracy of 0.9647	Cassava leaf disease	Scalability and accessibility for smallholder farmers
(Sambasivam et al., 2025)	VGG19, InceptionV3, and Inception-ResNet-V2.	Accuracy of 89.94%	Cassava leaf disease	Time-consuming, prone to error

Hassan and Maji (2022) presented a new CNN architecture optimized to identify plant diseases across various species, including cassava. Multi-scale convolutional filters and batch normalization were incorporated into the network to improve feature extraction and stability during convergence. The model achieved a total accuracy of 97.64% and a precision of 96.8% on the benchmark leaf datasets. Although the model performs well, it was trained on clean, balanced data, which limits its resilience to field noise, occlusion, and varying illumination. 

Latha et al (Rajasree et al., 2023). developed a faster, optimized Faster-RCNN model to identify CBSD. This method improved the accuracy of bounding-box hyperparameter optimization and reduced the false-positive object-detection error rate. The system achieved a mean average precision (mAP) of 92.6% and faster detection than a base Faster R-CNN. The large-labeled bounding-box data required by the model increased annotation costs and reduced its performance when exposed to novel disease variations or overlapping leaves.

Alejandro et al (Alejandro et al., 2025). used a deep learning approach with EfficientNet-B0 and large-scale image augmentation to detect cassava disease. The method exploited scaling up the depth, width, and resolution to achieve computational efficiency while maintaining accuracy. The accuracy and recall obtained in the experiment were 95.7% and 94.3%, respectively, indicating good generalization with a few parameters. However, the lightweight model misclassified visually similar diseases in some cases, specifically CMD and CBSD, suggesting that highly correlated features were not well discriminated.

Another salient feature establishment tactic suggested by Zhang et al (Zhang et al., 2024). is the fusion of deep features and spatial saliency maps for cassava disease recognition. This method had better interpretability and accuracy as it focused on discriminative lesion areas and eliminated background noise. The study’s accuracy was 96.84%, and its robustness across different lighting and occlusion conditions was demonstrated to be better. The model was not well-suited for real-time field deployment on low-power devices due to the computational overhead of the saliency extraction step.

Liu et al (Liu et al., 2022). proposed a Multi-Scale Fusion Model, based on EfficientNet and an attention mechanism, to classify cassava diseases. Multi-scale feature fusion enhanced the learning of local–global representations, and attention modules enhanced the focus on disease areas. This model achieved 98.03% accuracy and a 97.9% F1-score, beating traditional CNN baselines.

However, the network’s complexity increased training time and memory requirements, limiting its scalability and the ability to perform inference on the device in the field. Kalpana et al (Kalpana et al., 2024). proposed a deep learning-based approach to cassava leaf disease detection using data augmentation and CNN hyperparameter optimization. Their approach was intended to strike a balance between computational cost and performance, enabling a realistic implementation. Under normal testing conditions, the model achieved 96.25% accuracy and correctly differentiated diseases, but lacked a strong segmentation or feature-selection mechanism, leading to false positives on background-rich images and lower accuracy when tested on reduced data sets.

Farooqui et al (Farooqui et al., 2023). proposed a U-Net++ architecture for precise leaf disease segmentation, enhancing detection accuracy through multi-scale feature extraction and skip connections.

Bavana et al (Bavana et al., 2024). conducted a comprehensive study on machine learning methods in precision agriculture, analyzing algorithms for yield prediction, soil analysis, and crop health monitoring.

The key contributions of the research are as follows:

- To design a HyperCapsInception-ResNet-V2-CNN that combines Capsule Networks, Inception modules, and Residual Networks to improve the classification performance of cassava leaf images.
- To implement a data normalization procedure with adaptive Gaussian Otsu Thresholding (GOT) to enhance the visibility of disease regions and image quality.
- To introduce a Cascaded Canny Edge Segmentation (CCES) technique that is very effective in disease delimitation.
- To design an Optimal Spider Swarm Intelligence Technique (OSSIT) to reduce the number of features in question to the most pertinent ones, reducing redundant features, and enhancing the computational performance of the classification process.

The proposed method identifies several cassava leaf conditions, including CBB, CMD, CGM, CBSD, and normal leaf conditions. It obtains superior results based on 98.15% accuracy, a 97.22% F1-score, and 96.02% precision, and outperforms current deep learning models in detecting cassava disease. Our goal is to support the adoption of artificial intelligence (AI)-assisted precision agriculture, enabling early and accurate disease detection, minimizing crop losses, and enhancing cassava yield and sustainability.

This research is motivated by the necessity to improve the accuracy of disease detection and make precision farming possible with the help of an AI-based image analysis system that combines the following:

- Maximum Feature Selection with OSSIT to reduce dimensionality.
- A high-performance HyperCapsInception-ResNet-V2-CNN classifier captures complex leaf disease features.

This article aims to provide farmers and researchers with a scalable, reliable, and efficient diagnostic tool for sustainable cassava production and to contribute to global food security.

## 2 Proposed methodology

**Figure 1:**
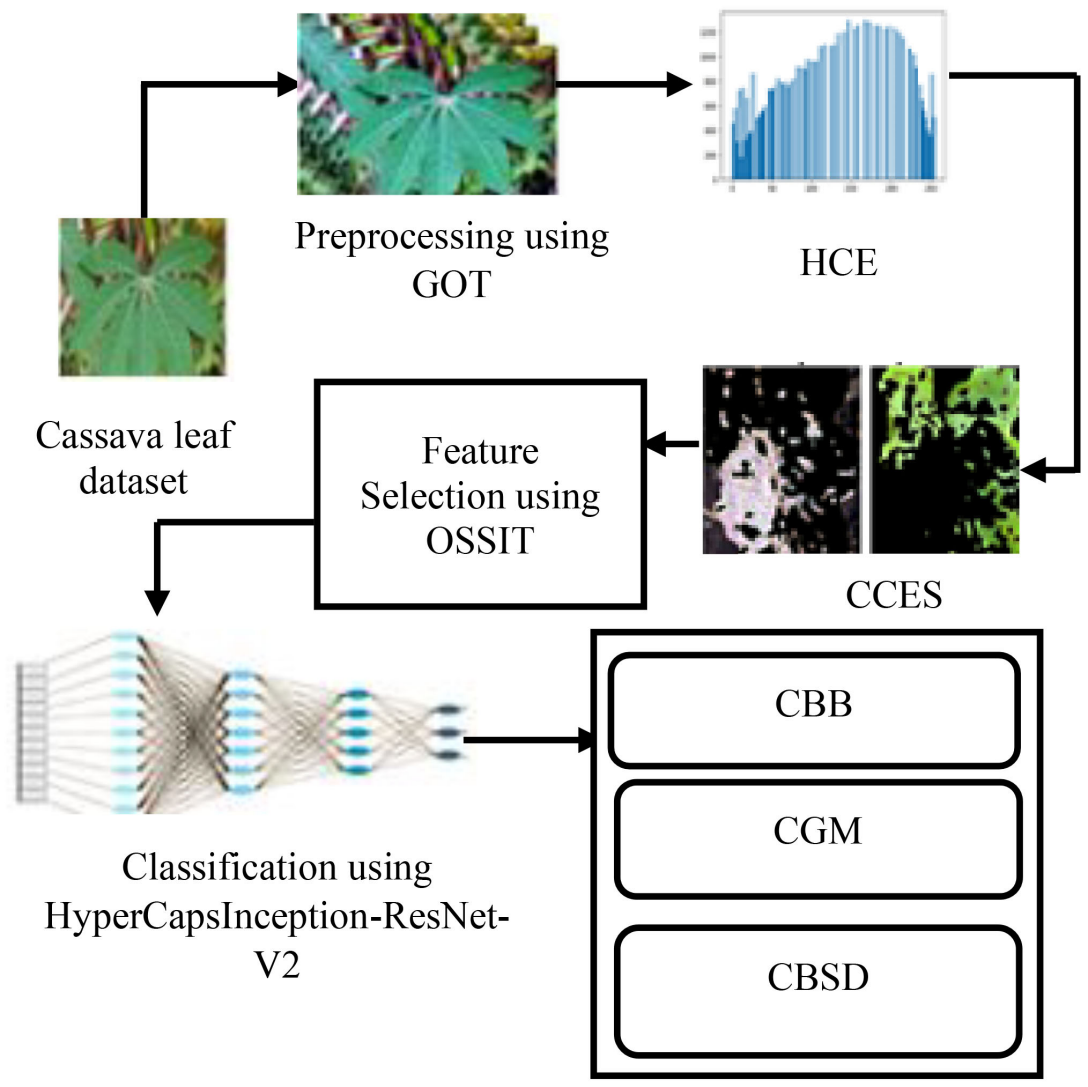
Proposed architecture diagram for cassava leaf disease detection.

This section identifies cassava leaf diseases using the proposed image-processing method. **Figure 1** describes the proposed architecture diagram for cassava leaf disease detection.

The adaptive GOT method is a mixture of the advantages of both Gaussian smoothing (to minimize noise) and Otsu global thresholding (to automatically separate the foreground from the background). This enhances contrast and normalization of the diseased areas prior to segmentation, so that there is equal intensity distribution among all the input samples. The suggested CCES executes edge detection in several fine steps, with both rough and satisfactory limits of infected areas being recorded. The OSSIT is a metaheuristic optimization algorithm based on the cooperative behavior of spiders and is a population algorithm. It is effective in choosing the best subsets of features by balancing between the exploration (diversity) and exploitation (intensification) of the feature space. The HyperCapsInception-ResNet-V2-CNN blends Inception modules to extract features at multiple scales, ResNet-V2 to avoid vanishing gradients and learn deep architectures, and Capsule Networks (CapsNets) to avoid the loss of spatial relations and rotational invariance in feature maps. The hybrid design is the unification of deep feature learning and spatial awareness, which allows for the more robust classification of diseases.

### 2.1 Gaussian Otsu thresholding preprocessing

**Figure 2:**
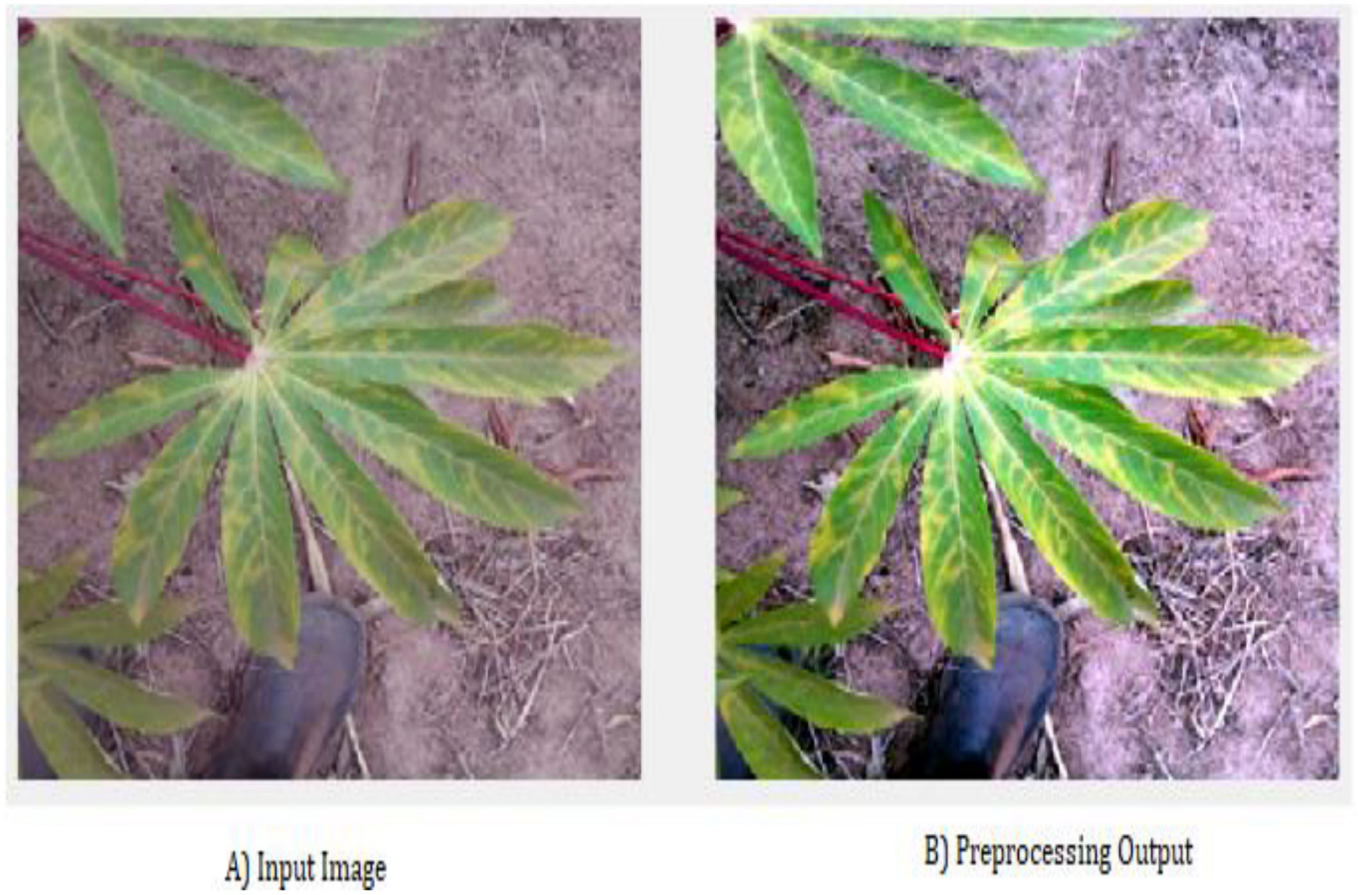
Preprocessing output.

Image preprocessing is the first step in enhancing the quality and usability of the Cassava Leaf dataset using Gaussian Otsu thresholding techniques. This step aims to improve image clarity, enabling more efficient pattern recognition and pixel color. Each pixel area accurately captures and preserves subtle yet significant features, such as leaf patterns. In **Figure 2**, the input consists of images of cassava leaves from a dataset in both normal and abnormal disease states. The preprocessing process enhances the contrast, ensuring that the image quality is suitable for further analysis. By refining low-visibility pixel points, preprocessing effectively classifies partially visible objects. It also eliminates undesirable distortions, thereby improving the essential features of the specific application being characterized.

In the first step, Pattern Identification, the vertical and horizontal orientations of the input image are analyzed. This analysis enables determining the optimal normalization level for converting grayscale images. The process analyzes changes in pixel intensity across rows and columns to identify distinctive cassava leaf patterns. By distinguishing between normal and abnormal leaf states, this granularity enables efficient feature extraction in subsequent processing stages.

(1) $$C_{i,j}=\sum_{\text{i}}{\text{S}}_{\text{i},\text{j}}=0\;$$

In Equation 1, the estimated coefficients $(C_{i,j})\;$ refer to input images, such as leaf shapes, and different values in the input image $\sum_{\text{i}}{\text{S}}_{\text{i},\text{j}}$. Here, i and j are the image’s pixels.

(2) $$\text{P}(\text{z})=(\;\frac{1}{\text{σ}\sqrt{2\text{π}}}){\text{e}}^{-{\left(\text{z}-\text{μ}\right)}^{2}-2{\text{ σ}}^{2}}$$

Equation 2 calculates the grayscale intensity  P(z). Here, μ (i, j) is the mean and  (σ) (i, j) is the standard deviation.

P(z) characterizes the gray level of the cassava leaf; the intensity of each pixel at coordinate (i, j) is affected by random noise, with 2σ (i, j) denoting the variance.

(3) $$x_{i,j}=\{\frac{{\text{b}}_{\text{i},\text{j}}\text{ with probability P}}{{\text{f}}_{\text{i},\text{j}}\text{ with probability }1-\text{P}}$$

Equation 3 calculates the noise level $\left(x_{i,j}\right)$, with each leaf having a different pixel at which the probability (P) is denoted by b (i, j). The minimum and maximum sizes of values are denoted by min and max, respectively, and the frequency (1 − P) is denoted by f (i, j).

### 2.2 Histogram color evaluation

A histogram is a visual representation of data that can be compared to a mathematical model. It divides an image into objects, regions, or subplots and processes each one independently. However, for cassava leaves, the entire gray-level range is processed simultaneously. The primary purpose of using a histogram for cassava leaves is to evaluate the output and select appropriate constraints. This involves analyzing the distribution of color values in images of cassava leaves, typically under controlled conditions. A histogram shows the frequency of different color intensities across an image’s color channels (such as red, green, and blue). It is used to assess leaf attributes, including health, nutrient levels, and disease status. For example, changes in the distribution of green hues may indicate variations in chlorophyll content. At the same time, abnormalities in the red and blue channels could signal the presence of pests or diseases. This analysis is crucial for the monitoring and early detection of issues in cassava crops.

**Figure 3:**
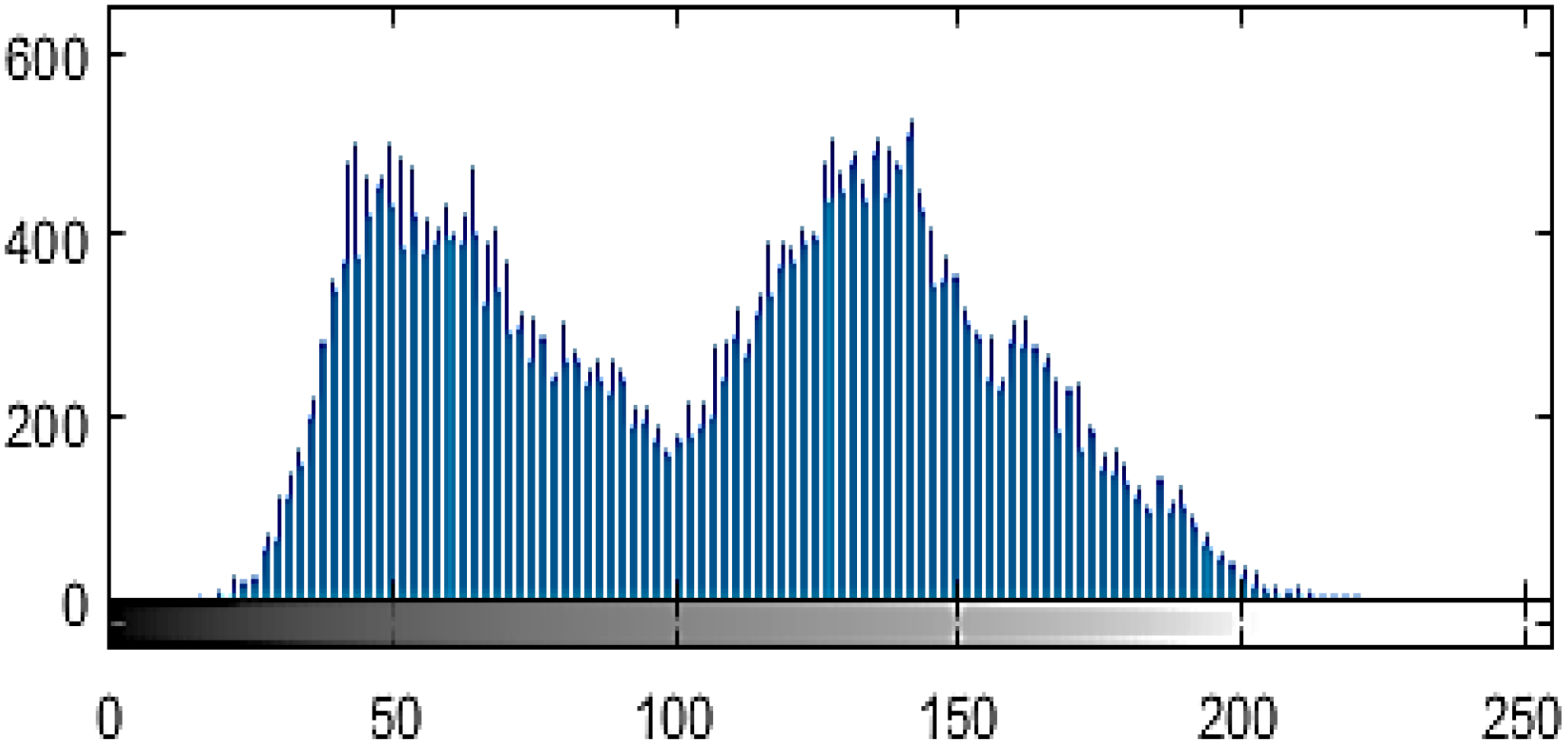
Histogram representation of cassava leaf.

**Figure 3** shows the output of the cassava plant leaf histogram image. Image contrast can be adjusted using histograms. By analyzing a histogram’s x-axis gray-level intensities, one can adjust an image’s contrast to suit specific requirements. Furthermore, histograms are frequently employed in image equalization, which produces high-contrast images by expanding the image’s gray-level intensities along the x-axis. Equation 4 estimates gray-level intensities.

(4) $$\text{ω}=\;\frac{\text{Range of data }}{\text{Number of Bins }}$$

ω is calculated based on the intensity range of the Maximum and the Minimum value. Choose the number of bins based on the dataset size and the preferred visualization. Frequency *f* (i, j) indicates the strength points of the pixels, which are evaluated in Equation 4.

(5) $${\text{hist}}_{\text{DN}}=\text{count }(\frac{\text{DN}}{\text{N}})$$

Equation 5 counts the number of frames in each “bin” and subtracts the image’s overall pixel count (N) from it. Here, DN denotes the intensity value of an individual pixel.

(6) $$\text{ρ}({\text{G}}_{\text{I}})=\;\frac{{\text{n}}_{\text{g}1}}{\text{N}}$$

Equation 6 was used to estimate the color enhancement between the healthy and disease-affected parts $\text{s of ρ}({\text{G}}_{\text{I}})$. Here, ${\text{n}}_{\text{g}1}$ represents the number of pixels in the image with a given intensity level.

### 2.3 Cascaded Canny Edge Segmentation

CCES uses the outer layer’s edge detection to improve edge detection. To increase edge precision and robustness, iterative pretreatment and refinement techniques are commonly used, especially for intricate visuals. The proposed method identifies the affected region by segmenting the leaves into distinct shapes, textures, and disease areas.

(7) $$\rho =\;S_{0}+\;s_{1}\;x+\;s_{1}\;x^{2}+\ldots \;\;+\;S_{u}\;x^{u}\;\;\;$$

The input pixel regularization value is x in this occurrence, and p represents the total number of pixels in the cassava leaf image. The complete collection of manipulated values by the training data { $S_{0}$ | a = 0, 1, 2, 255} for M = 255. To do this, the input x and output p are mathematically characterized as in Equation 7.

(8) $$k_{L}=\;m_{l}\;\frac{1}{\sum_{l=0}^{255}m_{l}},\;l=0,1,\;2$$

The image quality is evaluated before calculating the distance between two pixels. Total pixels *m_l_* {*l* = 0, 1, 2, 255} are evaluated for this using various regularization settings. The pixel value *k_L_* is finally described analytically in Equation 8.

(9) G(x, y) = ∥s(x + 1, y) − s(x − 1, y)∥2 + ∥s(x, y + 1) − s(x, y − 1)∥2

Equation 9 estimates the gradient computation stage, wherein s(x + 1, y) and  s(x − 1, y) denote the horizontal direction neighbor pixel values, and s( (x, y + 1)) and s (x, y − 1) present the vertical direction neighbor pixel values. A new “lab x, y” center is computed as the average of all vector pixels included in the cluster in order to divide the amount of clustering by the closest center of each pixel.

(10) $$T\;(x,y)=\;\frac{\left(1,\;\;if\;\;s(x,y)\;\geq T\right)}{\left(1,\;\;if\;\;s(x,y)\;<T\right)}$$

**Figure 4:**
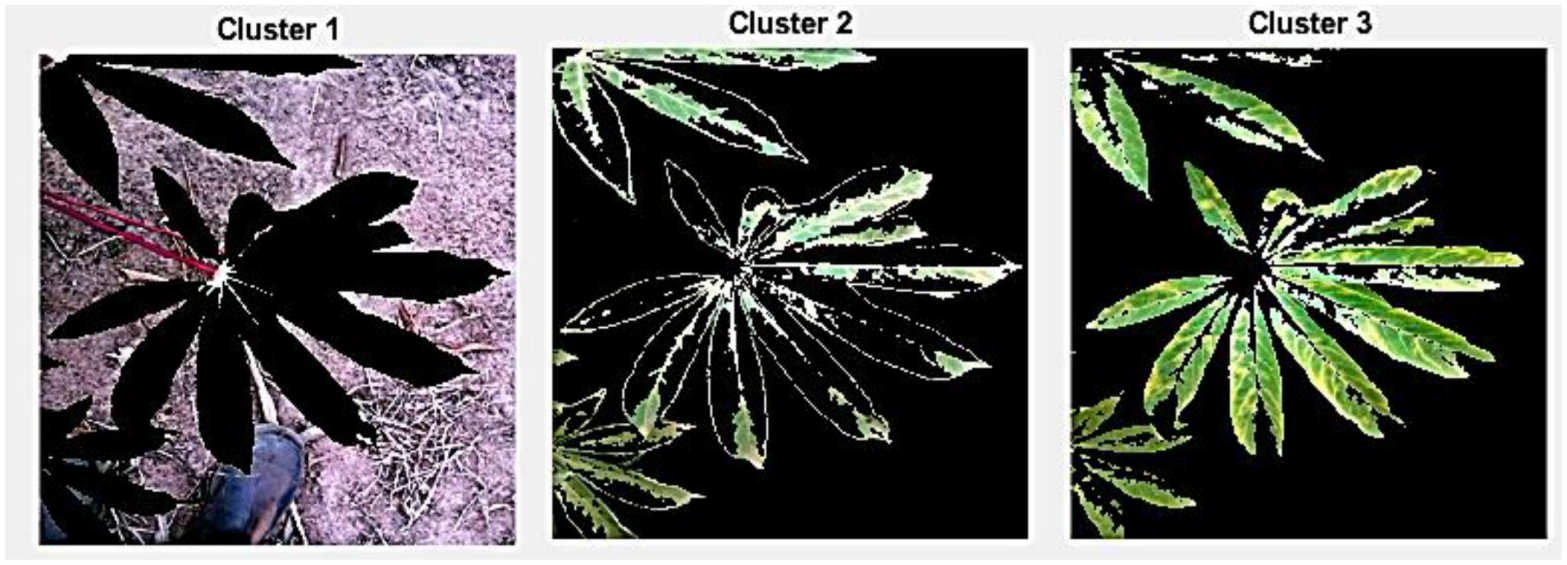
Canny edge segmentation output.

Equation 10 is used to determine the affected region T(x, y), where “*T*” stands for the threshold at pixel coordinate x, y; s (x, y) for the pixel value of the original input image in the “x, y” plane; and T (x, y) for the pixel values of the threshold image. **Figure 4** shows the output of the segmentation images.

### 2.4 Optimal Spider Swarm Intelligence Technique feature selection

The OSSIT is a selective optimization method inspired by spiders’ collective search behavior. It is designed to enhance the accuracy and efficiency of cassava plant leaf disease detection systems. OSSIT models the collaborative and competitive interactions among spiders to identify the most relevant features from high-dimensional datasets, such as those derived from leaf images. By iteratively optimizing a fitness function, OSSIT filters out redundant or irrelevant data, retaining only the features that significantly contribute to the accurate classification of diseases such as mosaic, brown streak, or bacterial blight. This method reduces computational complexity while enhancing machine learning models’ predictive capabilities, enabling the accurate, trustworthy detection essential for prompt cassava farming assistance. The technique demonstrates significant potential for agricultural applications, where early and accurate disease identification is vital for crop health and yield optimization. The following steps in the data analysis are based on the analysis below.

OSSIT algorithm steps

Step 1: Collect images of cassava leaf diseases and extract relevant characteristics, such as leaf shape, texture, and color histograms estimated by Equation 11.

(11) $${\text{S}}_{\text{i population}}=\;\frac{{\text{S}}_{\text{i}}-\text{min}(\text{s})}{\max(\text{x})-\text{min}(\text{s})}$$

Step 2: Fitness evaluation trains a classification model for each spider using the selected feature subset evaluated in Equation 12.

(12) Fitness=Max Accuracy

where ${\text{S}}_{\text{i}}$ generates the population of spider *i* from a fundamental component, and min(S) and max(S) are the minimum and maximum scores calculated in Equation 13:

(13) $$P_{\left(x,y\right)}R=\sum_{u=x-2}^{x+2}\sum_{v=y-2}^{y+2}a_{(u,v)R}I_{(u,v)R}$$

Step 3: Separate the male and female spiders as calculated in Equation 14. a (u, v) R is the linear estimate coefficient for the R channel for the position (u, v) R channel, with the difference (e (x, y) R) between the predicted pixel (P (x, y) R) and the central pixel (I (x, y) R) if calculating the weighted function in male and female spiders.

(14) $$e_{(x,y)R}=|I_{(x,y)R}-P_{(x,y)R}|$$

Step 4: The spider position is calculated using Equation 15 for the B channel (e(x, y) B) and the G channel (e(x, y) G). The spider position is set up in terms of the pixel (e (x, y)) in Equation 15:

(15) $$e_{\left(x,y\right)}=max\;(e_{(x,y)R},e_{(x,y)y},e_{(x,y)G})$$

Step 5: Male and female spiders consistently have high scores in the decision-making process evaluated in Equation 16. Decide on a feature importance threshold, e.g., G. mean total score ≥ 0 points 5 ≥ 0 points 5.

(16) $$x_{j}^{i}=\{\frac{1\;\;if\;x_{j}^{i}\;>\;Th}{0\;if\;\;x_{j}^{i}\;\leq \;Th}\}$$

The final step is to train the final disease detection model using the chosen features and evaluate its performance on a test dataset. The OSSIT optimizes feature subsets for feature selection by mimicking spider behavior, $x_{j}^{i}\;\;$. It begins with spiders (feature subsets) and evaluates their fitness using an objective function. This is a thresholding calculation that involves exploring and exploiting better solutions. This process maximizes model performance and chooses the most pertinent features at the end. Because of this, the weight value of dominant male spiders is greater than the male population’s median value. However, the weights of non-dominant male individuals are below the median.

**Figure 5:**
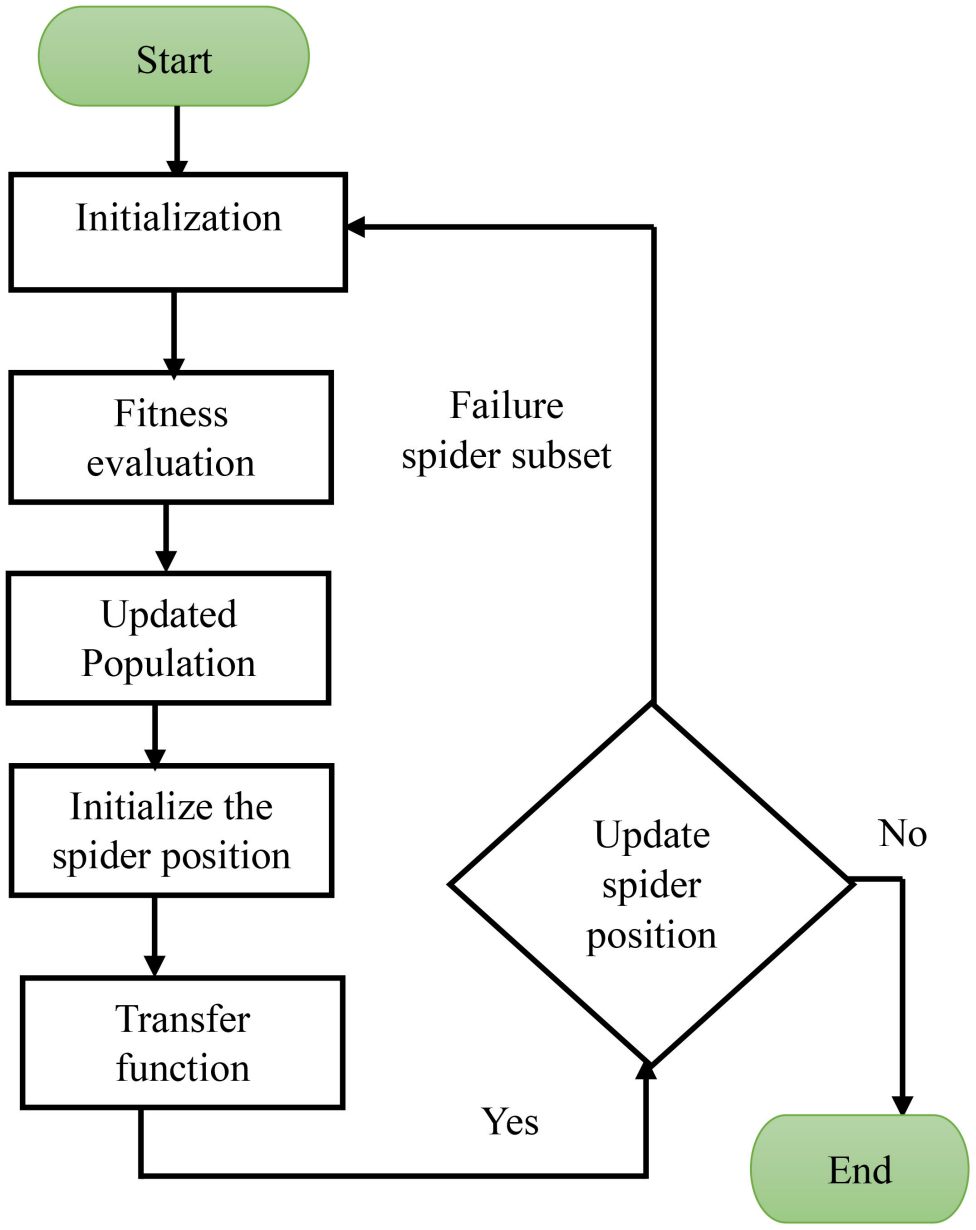
Flowchart for OSSIT feature selection. OSSIT, Optimal Spider Swarm Intelligence Technique.

**Figure 5** describes the workflow diagram of the OSSIT method for optimal feature selection of leaf disease detection. The process starts with initialization, which sets up the parameters, the spider population, and the starting positions. The effectiveness of each spider in choosing the best features is then evaluated using a fitness evaluation function. The population is updated based on the evaluation to include the most promising solutions, thereby focusing the search for pertinent features. To balance exploration and exploitation, the spiders’ positions are first initialized and then modified using a transfer function that simulates the dynamic movement of spiders in a swarm. Spiders that do not meet the requirements are placed in a failure subset during testing, which helps iterators update their positions.

The algorithm ends after this loop is completed and an ideal solution is found. In this iterative optimization process, the most pertinent features are selected to enable effective, precise disease detection.

**Table 2** describes the parameters of the OSSIT, which is used for feature selection in the detection of cassava plant leaf disease, along with their functions and initial experimental settings. The table provides a methodical framework for directing the algorithm’s behavior and optimizing its performance.

**Table 2:** OSSIT feature selection parameters.

Parameters	Description	Value
Population size	Number of agents (spiders) in the swarm	30
Max iterations	Maximum number of iterations to optimize features	100
Cognitive component (c1)	Weight for the individual’s best position	1.5
Social component (c2)	Weight for the best global location	2.0
Inertia weight (w)	Balances exploration and manipulation	0.9
Feature subset size	Size of the specific feature subset	10
Communication radius	Maximum distance for social interaction	5.0

OSSIT, Optimal Spider Swarm Intelligence Technique.

### 2.5 HyperCapsInception-ResNet-V2-CNN

A sophisticated hybrid deep learning architecture, the HyperCapsInception-ResNet-V2-CNN, is designed for challenging applications such as disease detection and image classification. It addresses important computer vision problems, such as multi-scale feature extraction, vanishing gradients, and spatial relationship preservation, by combining ResNet blocks, Inception modules, and Capsule Networks. The Cassava Plant Leaf Disease dataset was specifically tailored for this hybrid model, producing strong feature extraction and classification results.

HyperCapsInception-ResNet-V2-CNN combines two cutting-edge elements: Inception-ResNet-V2, a convolutional neural network that uses residual connections, and Inception modules to optimize deep feature extraction. With dynamic routing, CapsNets preserve spatial relationships and feature orientation while capturing hierarchical spatial structures. To extract rich hierarchical features, the architecture starts with the Inception-ResNet-V2 module. A Capsule layer is then added to improve spatial awareness. A fully connected capsule layer performs the final classification, enabling reliable disease classification.

**Figure 6:**
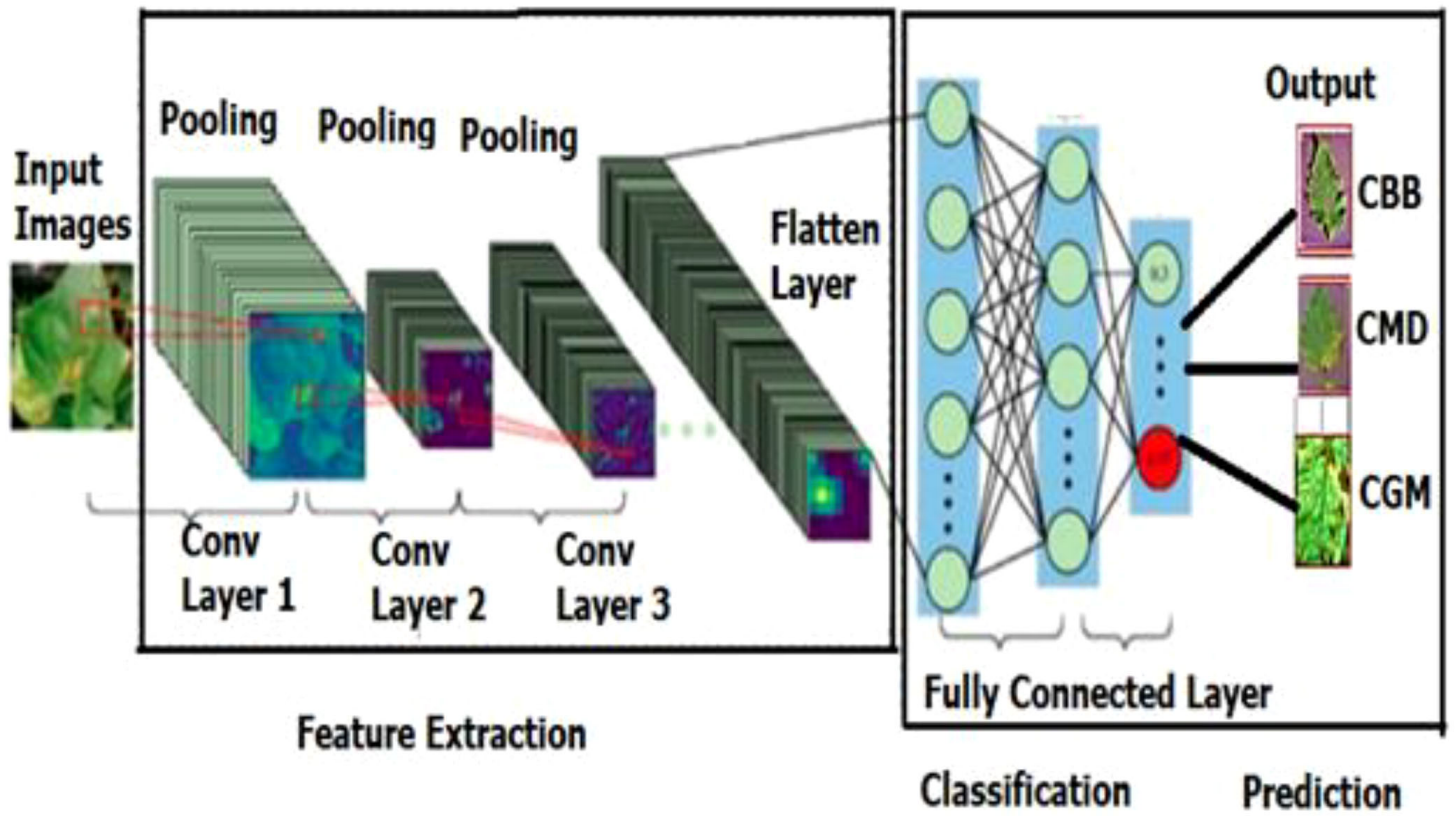
ResNet-V2-CNN architecture.

As shown in **Figure 6**, in the Cassava Plant Leaf HyperCapsInception-ResNet-V2-CNN, the input layer receives raw images of cassava leaves, which are typically resized and normalized for consistent processing. Convolutional layers apply filters to extract hierarchical features such as edges, textures, and patterns specific to cassava leaf diseases, leveraging Inception modules for multi-scale analysis and ResNet blocks for efficient gradient flow. By reducing the spatial dimensions while preserving important features, pooling layers, such as max pooling and average pooling, improve computational efficiency. To maintain spatial relationships and ensure reliable predictions even in the presence of variations in leaf pose or orientation, the output layer ultimately uses the Fitness layer to separate leaf classifications into distinct disease categories. Equation 17 evaluates the hidden features of cassava leaf disease.

(17) $${\text{p}}_{\text{i}}\in {\text{m}}_{\text{j}}{\text{ if f}}_{\text{ij}}\geq {\text{f}}_{\text{ix}}\;\forall \text{x},\text{ x }\in \text{m }$$

Here, ${\text{p}}_{\text{i}}$ denotes the feature point of i pixel, ${\text{m}}_{\text{j}}$ denotes the higher feature cluster (disease pattern), ${\text{ f}}_{\text{ij}}$ denotes feature strength, ${\text{f}}_{\text{ix}}$ denotes the activation function, and x  denotes the index of all possible clusters. The model can effectively converge on deeper networks, including ResNet architectures, which mitigate vanishing gradients and enable efficient gradient flow during backpropagation. These modules, which are intended for multi-scale analysis, improve the network’s ability to identify a wide range of features, from large textures to fine edges. To classify cassava leaves with different poses or orientations, Capsule Networks are essential because their layers maintain rotational invariance and spatial hierarchies.

Each pixel ${\text{p}}_{\text{i}}\;$ is regarded in this context as a cell “i” that is connected to k distinct cells $m_{1}$, $m_{2}$, and $m_{k}$. With a total of k classes, each m denotes a specific class. An ant is positioned at each cell i, and, using pheromone and heuristic values, the next cell k is chosen from the possible ${\text{m}}_{\text{j}}\;$ options. The pheromone function is defined based on the classes given to nearby pixels,; Equation 17 properly defines a pixel “i” that belongs to class “j”. The pheromone weighting function, Gaussian-based probability estimation, and final pixel classification are calculated using Equations 18–21).

Algorithm steps

Input: Extracted feature set

Step 1: Build feature vectors.

Construct the feature vectors $E_{1}$, $E_{2}$, and $E_{3}$, where *E*_1_ is the shape, *E*_2_ is the color, and *E*_3_ is the texture.

Step 2: Apply pattern classification.

Train each class of $E_{1}$, $E_{2}$, and $E_{3}$,

(18) $${\text{σ}}_{\text{ik}}=\sqrt{\sum_{\text{v}=1}^{\text{n}}({\text{V}}_{\text{i}}(\text{v})-{\text{CV}}_{\text{k}}(\text{v}){)}^{2}}$$

In Equation 18, pixel j is determined by assigning an angle α  to each cell. Compute the centroid vector ${\text{CV}}_{\text{k}}\;$ for every cell in the neighborhood ${\text{N}}_{\text{k}}$ of radius R for every cell K within a neighborhood of radius r around cell i.

Step 3: Estimate the pheromone weighting function $\text{W }({\text{σ}}_{\text{ik}})$.

(19) $$W\;(\sigma_{ik})=(1+\frac{\delta_{\alpha }}{\sum_{u\epsilon N_{i}^{t}}W\;(\sigma_{iu})}$$

where $\delta_{\alpha }$ is the pheromone detection limit and $\beta_{k}$ is the phenomenon consideration at cell k.

Step 4: Class probabilities and Gaussian distance weighting are used to compute pixel-level probabilities.

(20) $${\text{p}}_{\text{xi }}=\;\frac{1}{\text{z}(\text{σ})}\sum_{\text{u}\epsilon {\text{N}}_{i}^{\text{t}}}\text{W}({\text{σ}}_{\text{iu}}).{\text{ G}}_{\text{σ}}|\text{x}-\text{y}|$$

where ${\text{G}}_{\text{σ}}$ is a Gaussian function with standard deviation and z(σ) is the normalization term.

Step 5: Determine the location of each leaf disease pixel in the final class.

(21) $$c(x)=\;max_{\sum_{\text{u}\epsilon {\text{N}}_{i}^{\text{t}}}\text{W }({\text{σ}}_{\text{iu}})}$$

Output: Cassava mosaic disease, cassava green mite disease, and cassava brown streak disease are identified using selective features.

The HyperCapsInception-ResNet-V2-CNN is a prime example of how hybrid deep learning architectures can address challenging agricultural problems. By integrating spatial relationship preservation, efficient gradient flow, and multi-scale feature analysis, the model achieves notable gains in accuracy and resilience.

It provides a scalable approach to disease management and agricultural monitoring when used for cassava leaf disease detection. To increase its usefulness in precision agriculture, future research could examine how well it adapts to different crops and disease types.

**Figure 7:**
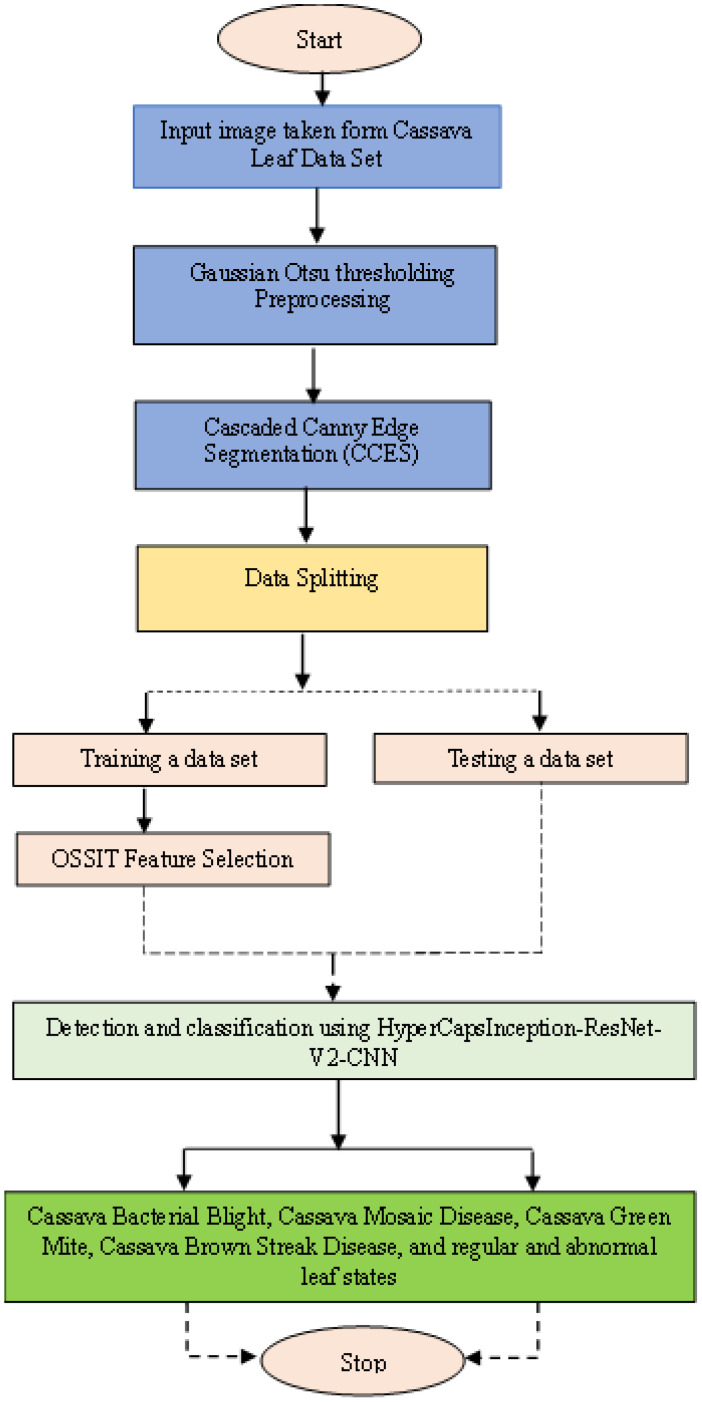
Overall flowchart of cassava leaf disease detection.

**Figure 7** presents a flowchart illustrating the application of OSSIT in conjunction with a neural network to analyze a dataset of cassava leaves. The preprocessing stage involves using Gaussian Otsu thresholding to improve image brightness and CCES for edge detection. This refined dataset is then used in OSSIT for feature selection, thereby enhancing the identification of significant features related to cassava brown streak disease. These features serve as the basis for training the HyperCapsInception-ResNet-V2-CNN model, enabling it to accurately classify diseases based on patterns. The model is then tested to evaluate its performance, completing the workflow for performance metrics. The dataset consists of images of cassava plant leaves from four disease categories and healthy leaves (CMD and CBB). To address class imbalance, data augmentation techniques such as flipping, rotation, and brightness adjustment are used during preprocessing. Additionally, normalization is applied to standardize the input features. The model is trained using a categorical cross-entropy loss function and an Adam optimizer with an initial learning rate of 0.001. To prevent overfitting, a dropout layer with a rate of 0.5 is added during training.

## 3 Results and discussion

Experiments were conducted to identify and analyze the performance of cassava plant leaf diseases. The first step involved collecting the cassava plant leaf dataset and applying preprocessing techniques. The following section evaluates the effectiveness of deep learning techniques using the proposed HyperCapsInception ResNet-V2-CNN classification system. Performance analysis was based on the mathematical evaluation of metrics, including the number of input images. Furthermore, this section provides a comprehensive analysis of classification methods and approaches across different datasets. **Table 3** describes the simulation parameters for disease classification.

**Table 3:** Simulation parameters.

Parameter	Value
Dataset name	Cassava Leaf Disease Classification
Number of images	21,367
Training images	16,025
Testing images	5,342
Language	Python
Tool	Jupyter

**Table 4** describes the proposed method’s hyperparameter settings for cassava leaf disease classification.

**Table 4:** Proposed method’s hyperparameter settings.

Parameters	Values
Optimizer	Adam
Learning rate	0.001
Number of epochs	100
Batch size	128
Loss function	Categorical cross entropy
Filters	128, 64, 32
Dropout	0.5

### 3.1 Cassava Plant Leaf dataset description

**Figure 8:**
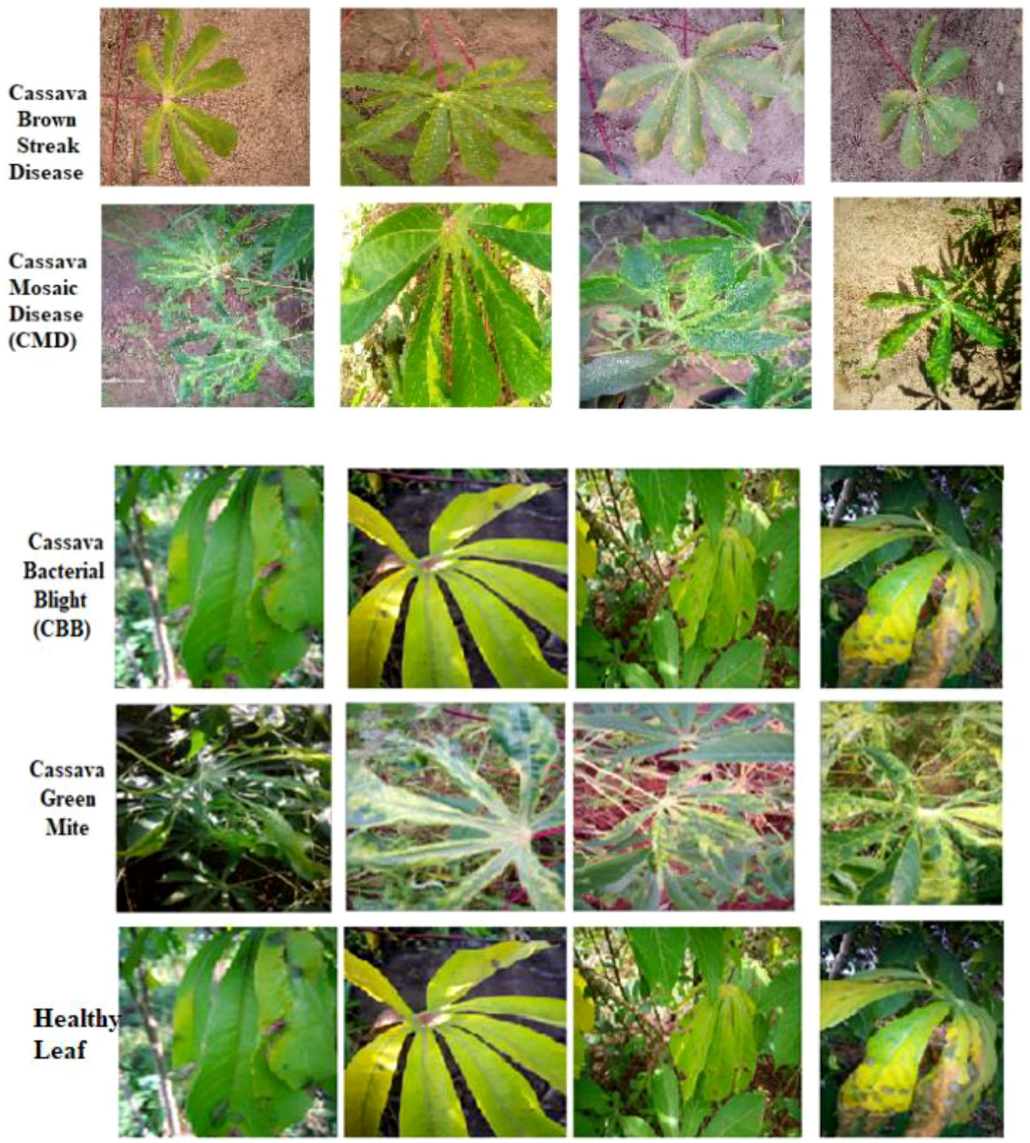
Examples of cassava plant leaf images.

The Cassava Leaf Disease Classification dataset, available on Kaggle, is a comprehensive collection of images designed to support the creation and evaluation of machine learning models for identifying diseases in cassava plants. This database contains five different folders, each describing a specific cassava disease: bacterial blight, brown streak disease, green mottle disease, healthy leaves, and mosaic disease. The dataset includes samples of healthy leaves and leaves with CBB, CBSD, CGM, and CMD. The images were organized by disease type, providing a variety of examples for training and testing classification models and facilitating the development of accurate cassava disease detection, as shown in **Figure 8**. The Cassava Leaf Disease Classification dataset is available on Kaggle and comprises 21,367 images. The images have an average resolution of 512 × 512 pixels. The data are split into training and test sets, enabling machine learning algorithms to be trained and tested to accurately detect diseases. The data are available for download from Kaggle: https://www.kaggle.com/datasets/nirmalsankalana/cassava-leaf-disease-classification.

**Figure 8** shows an example of the Cassava Plant Leaf dataset, consisting of images of leaves with diagnoses of CBB, CBSD, healthy leaves, and CGM, which are commonly employed to train models for plant disease detection. **Table 5** presents the distribution of images across five cassava leaf categories: CBB, CBSD, CGM, CMD, and Healthy leaves.

**Table 5:** Details of cassava leaf diseases.

Cassava leaf diseases	Training (75%)	Testing (25%)	Total
CBB	815	272	1,087
CBSD	1,642	547	2,189
CGM	1,789	597	2,386
CMD	1,933	644	2,577
Healthy leaves	9,846	3,282	13,128
Total	**16,025**	**5,342**	**21,367**

CBB, cassava bacterial blight; CMD, cassava mosaic disease; CGM, cassava green mite disease; CBSD, cassava brown streak disease.

Bold values represent the highest (best) performance.

**Table 5** provides the data for classifying cassava leaf diseases into the following categories: cassava bacterial blight, mosaic disease, green mite disease, and brown streak disease.

### 3.2 Experiment evaluation

The evaluation confusion matrix in **Table 6** shows the classification results: true positive (TP) = 250, true negative (TN) = 240, false positive (FP) = 5, and false negative (FN) = 5. These values indicate the counts for correct and incorrect predictions across positive and negative classes.

**Table 6:** Confusion matrix calculation.

Predicted class	True positive (TP)	True negative (TN)
Predicted positive	250	5
Predicted negative	5	240

#### 3.2.1 Precision

Precision indicates the proportion of predictions that are truly accurate when they are classified as positive. It is crucial when false positives are expensive and the goal is to reduce false alarms, as shown in Equation 22.

(22) $$\text{Precision}=\frac{\text{TP}}{\text{TP}+\text{FP}}$$

$$\text{Precision}=\frac{250}{250+5}=98\%$$

#### 3.2.2 F1-score

The F1-score provides a single metric that balances precision and sensitivity. When one class has substantially fewer samples than the other, it is beneficial for unbalanced datasets. The F1-score formula is presented in Equation 23.

(23) $$\text{F}1=\frac{TP}{TP+\frac{1}{2}(FP+FN)}\;$$

$$\text{F}1\text{ score}=\frac{0.9804\times 0.9804}{0.9804+\;0.9804}=97.22\%$$

#### 3.2.3 Accuracy

The overall correctness of both positive and negative predictions is measured using accuracy, as shown in Equation 24.

(24) $$\text{Accuracy}=\frac{\text{TP}+\text{TN}}{\text{TP}+\text{FP}+\text{TN}+\text{FN}}\;$$

$$\text{Accuracy}=\frac{250+240}{500}=98.15\%$$

#### 3.2.4 Sensitivity

Sensitivity is crucial when false negatives (missing positive cases) are a greater concern because it measures the model’s ability to identify actual positive cases, as shown in Equation 25.

(25) $$\text{Sensitivity}=\frac{\text{TP}}{\text{TP}+\text{FN}}\;\;\;\;\;\;\;\;\;\;\;\;\;\;\;\;\;$$

$$\text{Sensitivity}=\frac{450}{450+50}\;\;\;\;\;\;\;\;\;=\;97.96\;\%$$

#### 3.2.5 Specificity

Specificity quantifies the model’s ability to correctly detect negative cases, and it is estimated using Equation 26.

(26) $$\text{Specificty}=\frac{\text{TN}}{\text{TN}+\text{FP}}$$

$$\text{Specificty}=\frac{489}{11+489}\;\;\;\;\;\;\;\;\;=\;97.55\%$$

**Figure 9:**
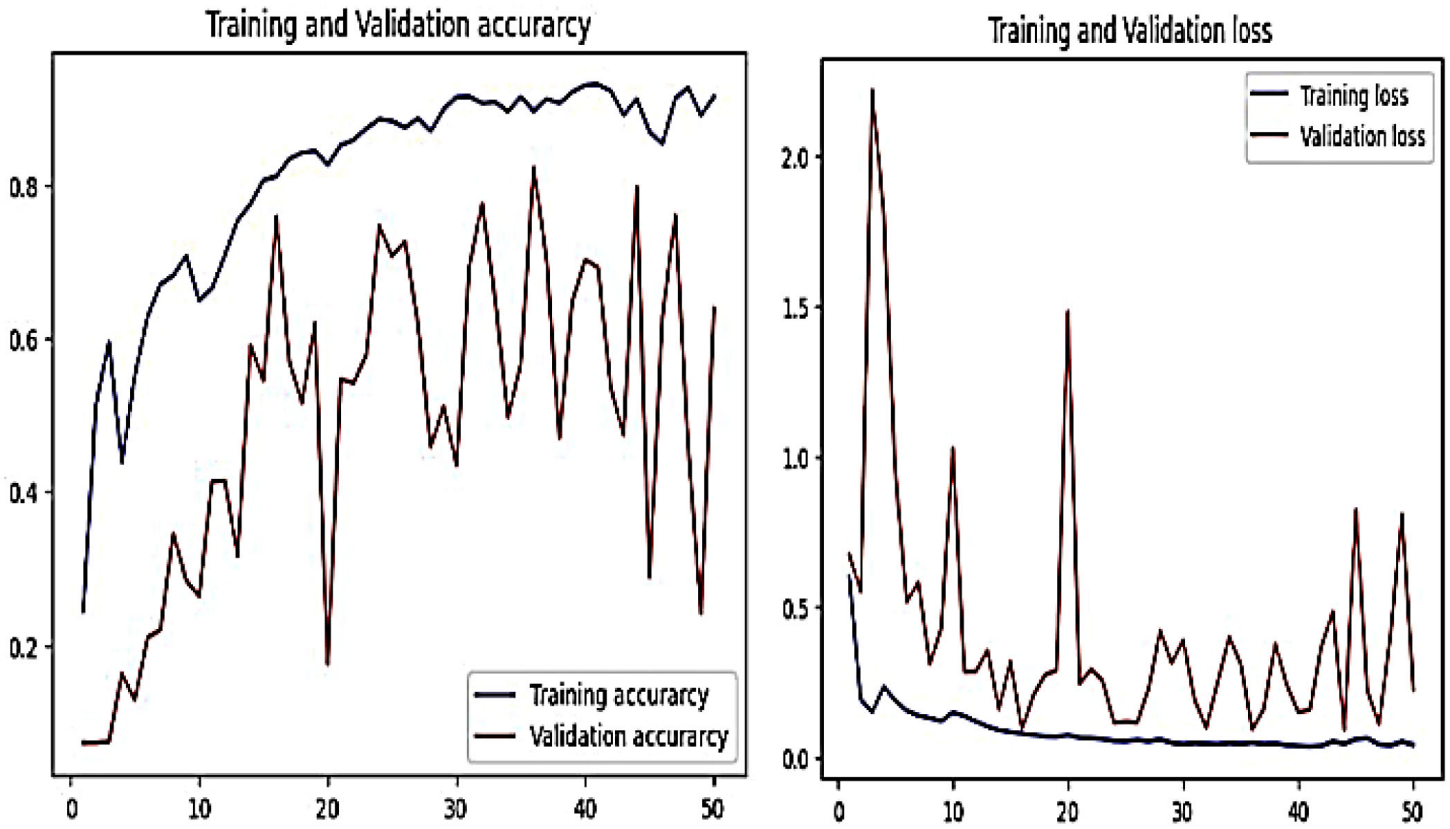
Graph of training and validation for accuracy and loss performance.

**Figure 9** shows the training and validation performance for accuracy and loss in disease detection. Even after 50 epochs, the CNN’s performance is worse than that of the recommended model after only 25 epochs. The pooling layer receives 896 parameters from 32 filters, using the 256x256-sized input from layer 1. With 16 filters and an input size of 85 × 85, the second layer produces 4,624 parameters. After that, the features proceed to the pooling layer and then to the flattened and dense layers. The total number of trainable parameters is much lower (231,347).

**Table 7** summarizes the performance metrics for a dataset that is split into training, validation, and testing sets. The table shows the number of samples, mean squared error (MSE), and error rate, with testing showing the highest error rate, indicating potential overfitting or differences in the data distribution.

**Table 7:** Performance of the proposed method’s mean squared error and normalized error.

Dataset	Number of sample images	MSE	Error
Training	160	1.85024 $e^{-1}$	24.281 $e^{-1}$
Validation	40	1.9404 $e^{-1}$	26.666 $e^{-1}$
Testing	40	2.0666 $e^{-1}$	35.555 $e^{-1}$

**Table 8** presents the classification performance of the HyperCapsInception-ResNet-V2-CNN model. A strong but somewhat diminished generalization was indicated by testing, with slightly lower accuracy (95.54%) and higher loss (0.12), whereas training shows the highest accuracy (98.65%) and lowest loss (0.10). The model successfully prevents overfitting due to the narrow gap between the training and validation/testing sets.

**Table 8:** Accuracy and loss analysis of the proposed method.

HyperCapsInception-ResNet-V2-CNN performance analysis		
Accuracy %	Training	98.65%
	Validation	96.23%
	Testing	95.54%
Loss %	Training	0.10
	Validation	0.11
	Testing	0.12

### 3.3 Performance analysis

This analysis compares existing classification techniques, such as EfficientNetB3, AlexNet, Faster R-CNN, and InceptionV3, with the proposed HyperCapsInception-ResNet-V2-CNN, evaluating metrics such as precision, recall, and the F1-score.

Cassava bacterial blight, cassava mosaic disease, cassava green mite disease, and cassava brown streak disease are among the cassava-specific diseases that, along with regular and abnormal leaf states, can be identified using the performance metrics shown in **Table 9**. Precision, sensitivity, specificity, accuracy, and F-measure are among the metrics that evaluate how well the diagnostic techniques work for each illness. Cassava green mite exhibited the highest sensitivity (91.47%) and specificity (92.19%), while the maximum precision (88.35%) and F-measure (0.95) were noted for regular and abnormal leaf states. Accuracy metrics for these diseases ranged from 84.11% to 88.35%, indicating that, overall, the detection methods are highly reliable and accurate.

**Table 9:** Performance metrics based on different categories in cassava diseases.

Categorization of cassava leaf diseases	Precision (%)	Sensitivity (%)	Specificity (%)	Accuracy (%)	F-Measure
Cassava bacterial blight	84.11	84.44	90.12	88.58	0.94
Cassava mosaic disease	85.34	89.05	90.45	89.55	0.94
Cassava green mite disease	86.70	91.47	92.19	90.31	0.92
Cassava brown streak disease	84.55	88.55	90.24	88.50	0.94
Regular and abnormal leaf states	88.35	91.05	91.56	90.10	0.95

The performance metrics of the various approaches are shown in **Table 10** for accuracy, sensitivity, specificity, precision, and F-measure. The techniques that were assessed were HyperCapsInception-ResNet-V2-CNN, AlexNet, Faster-RCNN, InceptionV3, and EfficientNetB3. The proposed HyperCapsInception-ResNet-V2-CNN method achieved an accuracy of 98.15%, an F-measure of 97.22%, a precision of 96.02%, a sensitivity of 97.96%, and a specificity of 97.55%. Previous approaches performed worse than the proposed system.

**Table 10:** Comparison of performance for cassava disease detection.

Methods	Precision (%)	Sensitivity (%)	Specificity (%)	Accuracy (%)	F-Measure (%)
EfficientNetB3 (Riyadi et al., 2024)	96.10	94.50	97.40	96.20	92.20
AlexNet (Chilakalapudi and Jayachandran, 2024)	93.40	92.20	95.70	92.80	94.30
Faster-RCNN (Rajasree et al., 2023)	90.30	87.80	91.60	89.50	95.05
InceptionV3 (Sambasivam et al., 2025)	95.20	93.33	96.20	94.20	96.13
HyperCapsInception-ResNet-V2-CNN	96.02	97.96	97.55	98.15	97.22

**Figure 10:**
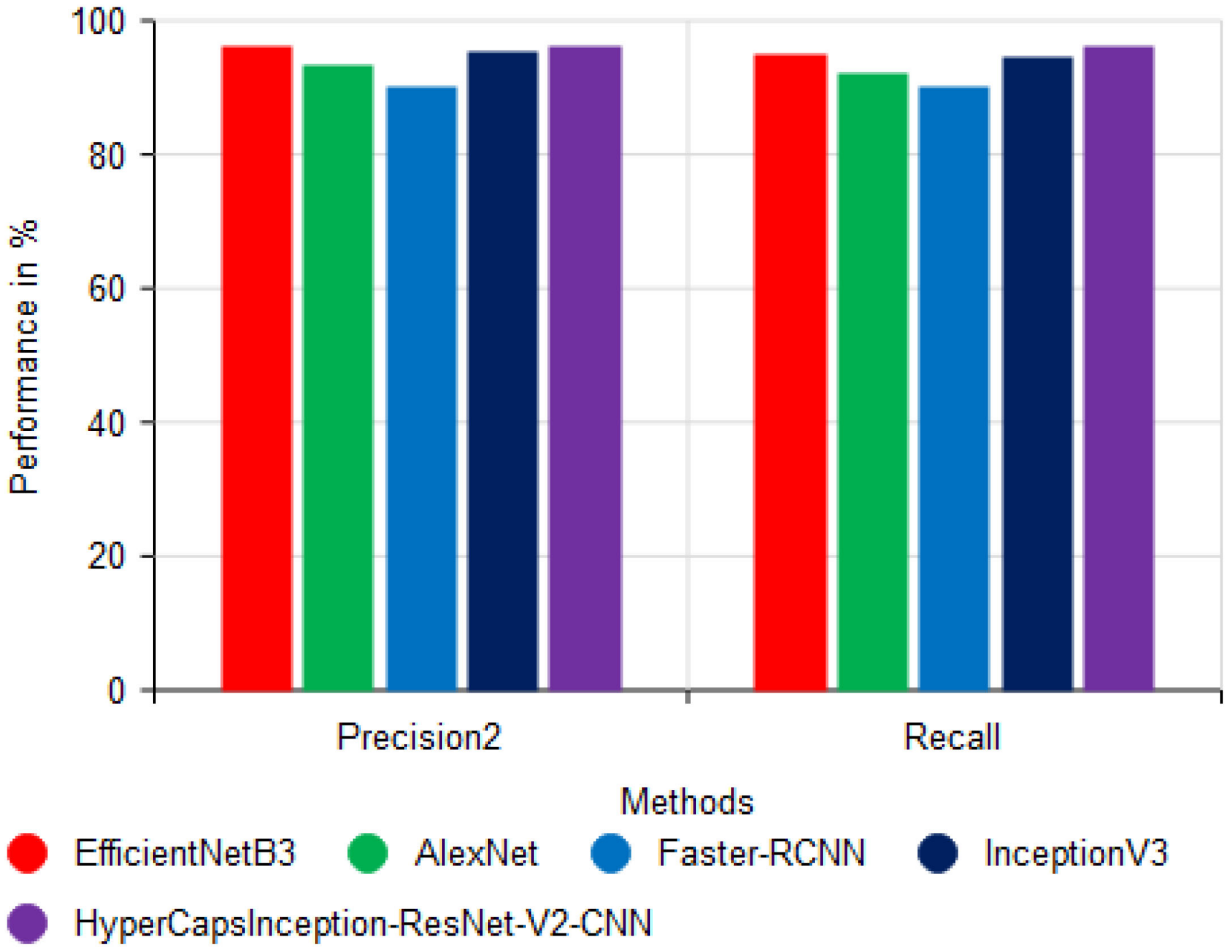
Comparison of precision and recall performance.

The sensitivity and specificity of the various models are compared in **Figure 10**, which demonstrates that the suggested HyperCapsInception-ResNet-V2-CNN method achieved a precision of 96.02% and a recall of 96.18%. Similarly, previous methods performed worse.

**Figure 11:**
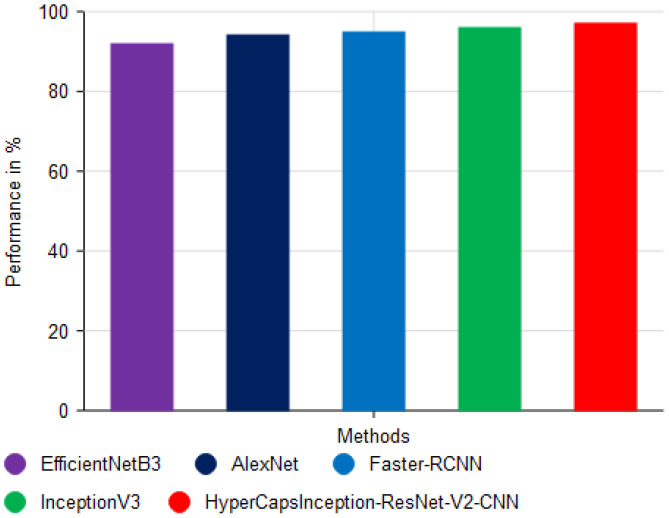
Analysis of F1-score performance.

**Figure 11** shows the performance in terms of F1-score (%) of different deep learning approaches to classify cassava disease with different proposed models, and it can be observed that the HyperCapsInception-ResNet-V2-CNN model is the most effective. The x-axis indicates the various classification methods, whereas the y-axis shows their performance as percentages. The EfficientNetB3 method achieved an F1-score of 92.20%, which is very good, as it leverages feature extraction and time-series modeling.

The AlexNet (Chilakalapudi and Jayachandran, 2024) approach achieved an accuracy of 94.30%, which is considered moderate and shows limited ability to capture complex disease patterns in leaf images. The Faster-RCNN (Rajasree et al., 2023) approach with haste achieved an accuracy of 95.05% with the advantage of recurrent modeling, but it is marginally lower than the most effective models. The InceptionV3 approach also scored an accuracy of 96.13%, indicating the usefulness of deep convolutional networks for image-based classification, albeit without the improved feature optimization provided by the proposed model. The proposed HyperCapsInception-ResNet-V2-CNN method achieved the highest F1-score at 97.22%, indicating its greater ability to detect and categorize cassava leaf diseases accurately. This shows the benefit of integrating Capsule Networks, Inception modules, and ResNet-V2 for robust feature learning, particularly when coupled with optimal feature selection via OSSIT.

**Figure 12:**
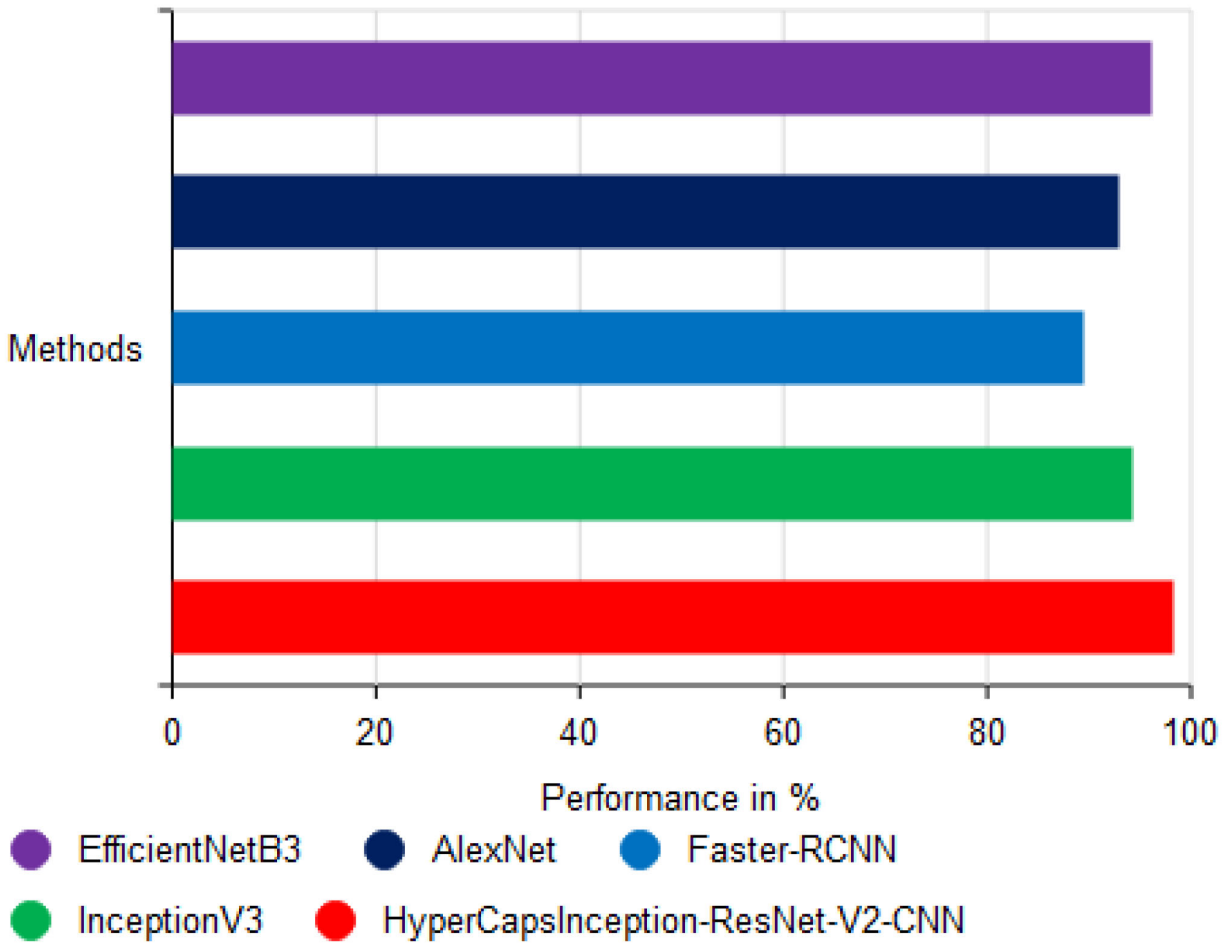
Analysis of accuracy performance.

**Figure 12** shows the accuracy (%) of different deep learning approaches for classifying cassava diseases using the proposed models, and it can be observed that the HyperCapsInception-ResNet-V2-CNN model is the most effective. The x-axis indicates the various classification methods, whereas the y-axis shows their performance as percentages. The EfficientNetB3 method achieved an accuracy of 96.2%, which is very good, as it uses feature extraction and time-series modeling. The AlexNet (Chilakalapudi and Jayachandran, 2024) approach achieved an accuracy of 92.8%, which is considered moderate and has limited ability to capture complex disease patterns in leaf images. The Faster-RCNN (Rajasree et al., 2023) approach achieved an accuracy of 89.5%, benefiting from recurrent modeling, but still performing marginally lower than the most effective models. The InceptionV3 approach scored an accuracy of 94.2%, demonstrating the usefulness of deep convolutional networks for image-based classification, though it lacks the enhanced feature optimization achieved by the proposed model. The proposed HyperCapsInception-ResNet-V2-CNN method achieved 98.15% accuracy, indicating greater ability to detect and categorize cassava leaf diseases. These results highlight the effectiveness of integrating Capsule Networks, Inception modules, and ResNet-V2 for robust feature learning, especially when combined with optimal feature selection through OSSIT.

**Figure 13:**
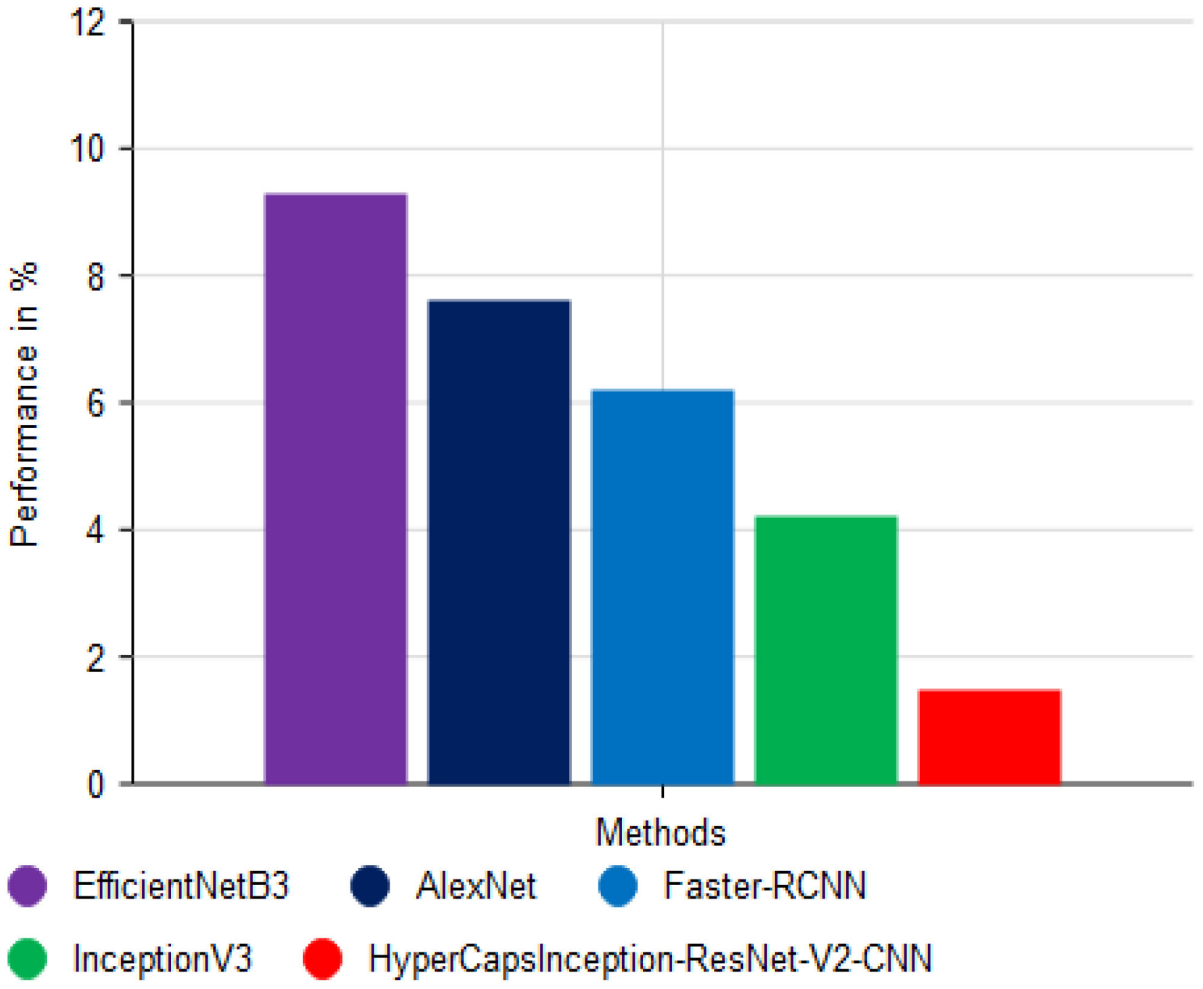
Comparison of error rate analysis.

**Figure 13** shows the error rate analysis of the different classification outputs, with the proposed HyperCapsInception-ResNet-V2-CNN achieving the lowest error rate of 1.5%, indicating its superior accuracy. In comparison, the EfficientNetB3 model error rate was 9.3%, the AlexNet method error rate was 7.6%, the Faster-RCNN method error rate was 6.2%, and the error rate for the InceptionV3 algorithm was 4.2%. This demonstrates the significant improvement in error minimization achieved by the proposed model.

**Figure 14:**
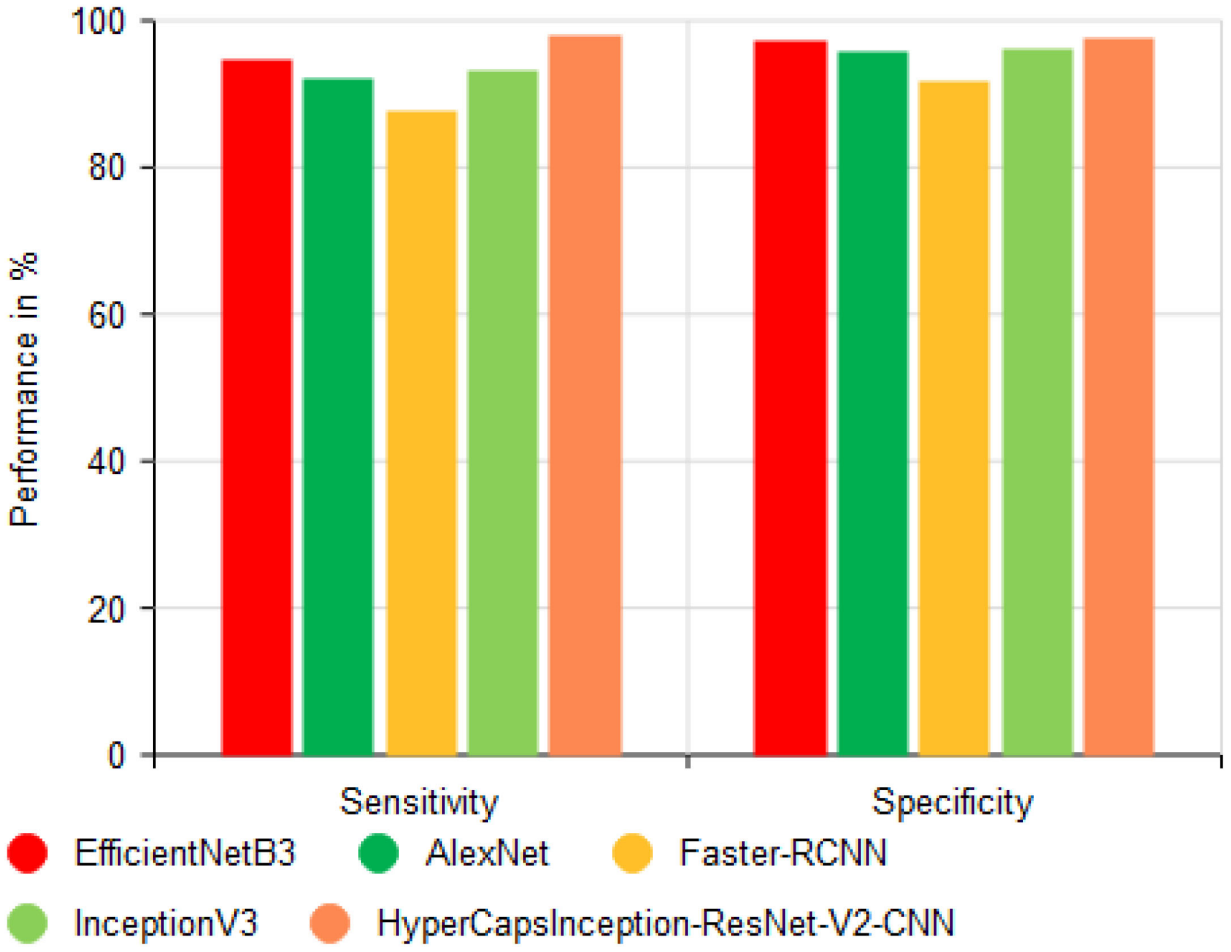
Comparison of sensitivity and specificity analyses.

As shown in **Figure 14**, the HyperCapsInception-ResNet-V2-CNN achieved the highest sensitivity (97.96%) and specificity (97.55%) among existing methods.

### 3.4 Discussion

The experimental data show that the proposed HyperCapsInception-ResNet-V2-CNN model consistently outperforms current deep learning models in the classification of cassava leaf diseases, as measured using F1-score and accuracy. The proposed model outperformed EfficientNetB3 (F1-score 92.20% and accuracy 96.20%), AlexNet (F1-score 94.30% and accuracy 92.80%), Faster-RCNN (F1-score 95.05% and accuracy 89.50%), and InceptionV3 (F1-score 96.13% and accuracy 94.20%), as indicated by the results in **Figures 11** and **12**. The combination of the three potent components of ResNet-V2, which guarantees deep residual learning and eliminates gradient vanishing to facilitate more efficient feature extraction, is the cause of the improved performance of the proposed model. Inception modules extract multi-scale spatial features and sophisticated patterns in leaf images. Capsule Networks, which invariably maintain spatial hierarchies and part–whole relations, improve the model’s ability to differentiate subtle differences among disease classes. Also, integrating optimal feature selection with OSSIT reduces irrelevant and redundant features, achieving higher classification accuracy and fewer false positives. All of these strategies, combined, enable the given model to effectively manage the complex changes in cassava leaf images, including overlapping disease symptoms, irregular leaf texture, and different illumination conditions. In comparison with them, the available approaches were found to be inadequate in their ability to learn these complexities: EfficientNetB3 has the advantage of sequential modeling and feature extraction, but lacks the strong spatial hierarchy learning of Capsule Networks. The depth of AlexNet and its ability to learn complex patterns are limited, leading to mediocre performance. Faster R-CNN exploits temporal connections at the expense of spatial feature representations in images, and thus, its F1-scores and accuracy are slightly lower. Subsequently, InceptionV3 is effective at extracting multi-scale features. However, it fails to optimize feature selection and relies on capsule-based hierarchical learning, which limits its performance compared to the proposed method. In addition, the proposed model has better validation and verification stability, lower training loss, and fewer false detections, demonstrating better generalization and lower overfitting. These findings verify that the hybrid combination of ResNet-V2, Inception, and Capsule Networks, along with OSSIT-based optimal feature selection, provides a strong and precise framework for cassava leaf disease classification. Overall, as discussed, the proposed approach yields the best classification results and helps overcome the limitations of current deep learning techniques, making it a promising solution for the automated detection of cassava disease in real-life agricultural applications.

## 4 Future scope

Despite the fact that the proposed HyperCapsInception-ResNet-V2-CNN model can be regarded as having excellent performance for cassava disease detection and classification, there are still a number of opportunities for future research. First, the study carried out was based on images that were taken under controlled lighting and background conditions, which is why future research could focus on the creation of a stronger model that can operate with real-time field images that were taken under different lighting, occlusion, and environmental conditions. The combination of multi-spectral and hyperspectral image information could also improve the ability of the model to detect minor disease signs that cannot be observed easily in RGB images.

The other avenue to explore is the implementation of lightweight architectures and model compression methods to allow the detection of diseases on edge devices and smartphones in real-time, thus assisting the farmers living in remote or low-resource settings. Moreover, it is possible to expand the model to a multi-crop disease classification system and enhance its scale and agricultural impact.

Furthermore, the integration of explainable AI (XAI) methods would enable users to see and understand the basis of disease predictions, making the system more transparent and reliable. Finally, the application of decision support or treatment recommendation modules to link the disease detection model could help make this strategy into an all-encompassing digital agriculture platform that supports the early diagnosis, monitoring, and treatment of plant health.

## 5 Conclusion

In conclusion, this study introduced a sophisticated artificial intelligence-driven image analysis system that is effective and precise in identifying cassava plant diseases. The proposed system combines adaptive preprocessing, optimal feature selection, and a deep hybrid classification model to address the shortcomings of existing cassava disease detection systems. The GOT technique was used to normalize the image data, improving contrast and eliminating noise. Meanwhile, the isolation of disease regions was enhanced by other methods, namely, Histogram Color Evaluation (HCE) and Iterative Clustering Fragmentation. Moreover, the CCES technique was successfully used to outline infection areas on the leaf, and the OSSIT technique was used to eliminate redundant and irrelevant feature dimensions, ensuring that the features are well represented. The proposed HyperCapsInception-ResNet-V2-CNN model achieved better classification performance than traditional deep learning architectures, including EfficientNetB3, AlexNet, Faster-RCNN, and InceptionV3. The results of the experiments showed that the proposed method was overall accurate (98.15%) and had better F1-scores and a considerable decrease in false detection rates. The results show that optimized feature extraction and a hybrid deep learning architecture improve the accuracy and robustness of cassava disease detection.

## Ethical and informed consent for the data used

The data used in this study have not been subjected to any ethics or consent approval by the authors.

## Data Availability

The original contributions presented in the study are included in the article/supplementary material. Further inquiries can be directed to the corresponding author.
